# Diagnostic accuracy of quantitative dual-energy CT-based volumetric bone mineral density assessment for the prediction of osteoporosis-associated fractures

**DOI:** 10.1007/s00330-021-08323-9

**Published:** 2021-10-29

**Authors:** Leon D. Gruenewald, Vitali Koch, Simon S. Martin, Ibrahim Yel, Katrin Eichler, Tatjana Gruber-Rouh, Lukas Lenga, Julian L. Wichmann, Leona S. Alizadeh, Moritz H. Albrecht, Christoph Mader, Nicole A. Huizinga, Tommaso D’Angelo, Silvio Mazziotti, Stefan Wesarg, Thomas J. Vogl, Christian Booz

**Affiliations:** 1 Clinic of the Johann-Wolfgang Goethe Universität Frankfurt, Theodor-Stern-Kai 7, 60590 Frankfurt am Main, Germany; 2 Department of Diagnostic and Interventional Radiology, University Hospital Frankfurt, Frankfurt am Main, Germany; 3 Department of Biological Psychology, Vrije University Amsterdam, Amsterdam, The Netherlands; 4 Department of Biomedical Sciences and Morphological and Functional Imaging, University Hospital Messina, Messina, Italy; 5 Fraunhofer IGD, Darmstadt, Germany

**Keywords:** Bone density, Osteoporosis, Osteoporotic fractures, Bone diseases, Metabolic, CT dual-energy computed tomography

## Abstract

**Objectives:**

To evaluate the predictive value of volumetric bone mineral density (BMD) assessment of the lumbar spine derived from phantomless dual-energy CT (DECT)-based volumetric material decomposition as an indicator for the 2-year occurrence risk of osteoporosis-associated fractures.

**Methods:**

L1 of 92 patients (46 men, 46 women; mean age, 64 years, range, 19–103 years) who had undergone third-generation dual-source DECT between 01/2016 and 12/2018 was retrospectively analyzed. For phantomless BMD assessment, dedicated DECT postprocessing software using material decomposition was applied. Digital files of all patients were sighted for 2 years following DECT to obtain the incidence of osteoporotic fractures. Receiver operating characteristic (ROC) analysis was used to calculate cut-off values and logistic regression models were used to determine associations of BMD, sex, and age with the occurrence of osteoporotic fractures.

**Results:**

A DECT-derived BMD cut-off of 93.70 mg/cm^3^ yielded 85.45% sensitivity and 89.19% specificity for the prediction to sustain one or more osteoporosis-associated fractures within 2 years after BMD measurement. DECT-derived BMD was significantly associated with the occurrence of new fractures (odds ratio of 0.8710, 95% CI, 0.091–0.9375, *p* < .001), indicating a protective effect of increased DECT-derived BMD values. Overall AUC was 0.9373 (CI, 0.867–0.977, *p* < .001) for the differentiation of patients who sustained osteoporosis-associated fractures within 2 years of BMD assessment.

**Conclusions:**

Retrospective DECT-based volumetric BMD assessment can accurately predict the 2-year risk to sustain an osteoporosis-associated fracture in at-risk patients without requiring a calibration phantom. Lower DECT-based BMD values are strongly associated with an increased risk to sustain fragility fractures.

**Key Points:**

*•Dual-energy CT–derived assessment of bone mineral density can identify patients at risk to sustain osteoporosis-associated fractures with a sensitivity of 85.45% and a specificity of 89.19%.*

*•The DECT-derived BMD threshold for identification of at-risk patients lies above the American College of Radiology (ACR) QCT guidelines for the identification of osteoporosis (93.70 mg/cm* ^*3*^ *vs 80 mg/cm* ^*3*^ *).*

## Introduction

Individuals suffering from osteoporosis are at increased risk to sustain fragility fractures. In aging populations, the incidence of osteoporosis and the personal and economical burdens of osteoporosis-associated fractures are expected to rise significantly [1].

Osteoporotic fractures are a major cause of immobility and frailty among aging groups. While management and treatment of fractures account for two-thirds of the costs associated with osteoporosis, pharmacological prevention only accounts for ~ 5% [1, 2]. Therefore, more accurate and earlier identification of individuals suffering from and individuals at risk to develop osteoporosis is necessary to counteract the progressive destruction of bone architecture and reduce the associated social and economic burden.

In 1994, dual x-ray absorptiometry (DXA) was proposed by the WHO as diagnostic gold standard for the diagnosis of osteoporosis [3]. However, studies have demonstrated that DXA measurements do not accurately assess the risk for osteoporosis-associated fractures [4]. A major limitation of DXA is that areal BMD of the entire vertebral body is measured, which is prone to distortions caused by variations in body composition, overlying soft tissue, and vascular calcification [5–7].

The use of quantitative computed tomography (QCT) can overcome some of the previously mentioned limitations by assessing volumetric BMD of trabecular bone and shows superior sensitivity for the prediction of osteoporosis-associated fractures. However, conventional QCT measurements require the use of calibration phantoms and, generally, cannot be applied retrospectively. Therefore, the problem of underdiagnosing osteoporosis is not addressed [8–11].

The growing number of CT scans makes the widespread use of opportunistic BMD assessment a promising approach for the detection of osteoporosis. Different methods have been suggested for opportunistic BMD assessment, such as simple Hounsfield unit (HU) measurements [12–14]. However, it has been shown that HU-based BMD measurements share most weaknesses of DXA such as distortion by changes in body composition and overlying tissue [15].

Material differentiation in DECT can provide novel relevant information for different musculoskeletal applications compared to conventional CT [16–18]. Recently, a DECT-based postprocessing algorithm, which permits phantomless volumetric BMD assessment of lumbar trabecular bone, has been evaluated, showing superior results for the detection of osteoporosis compared to HU measurements as well as strong correlation with bone strength in human cadaver vertebrae specimens [17–20].

We hypothesized that the high diagnostic accuracy of phantomless volumetric DECT BMD assessment based on material decomposition may also enable accurate predictions for the incidence of osteoporosis-associated fractures. Therefore, the purpose of this study was to evaluate the predictive value of BMD measurements derived from phantomless DECT-based volumetric material decomposition of the lumbar spine as an indicator for the 2-year occurrence risk of osteoporosis-associated fractures.

## Materials and methods

This retrospective study was approved by the institutional review board. The requirement to obtain written informed consent was waived.

### Patient selection and study design

**Fig. 1:**
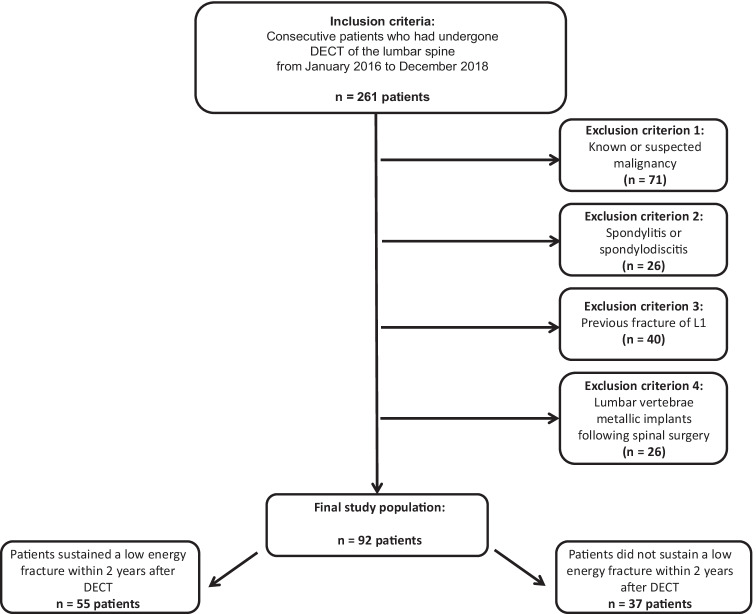
STARD (Standards for Reporting of Diagnostic Accuracy Studies) flow chart of patient inclusion

Patients were considered for inclusion in this study if they had undergone non-contrast third-generation dual-source DECT of the lumbar spine between January 2016 and December 2018 (Fig. 1). Exclusion criteria were suspected or known malignancy, spondylitis or spondylodiscitis, lumbar vertebrae with metallic implants following spinal surgery, previous fracture of L1, and severe deformity of L1.

Fractures of the following body regions were regarded as osteoporosis-associated if no adequate trauma was present: spine, proximal humerus, distal radius, and proximal femur. In this context, 55 patients sustained an osteoporosis-associated fracture following a low-energy trauma within 2 years.

### CT protocol

CT studies were performed on a third-generation dual-source CT system in dual-energy mode (SOMATOM Force; Siemens Healthineers). Both x-ray tubes operated at different kilovoltage settings (tube A: 90 kVp, 180 mAs; tube B: Sn150 kVp [0.64-mm tin filter], 180 mAs). Image series were collected in craniocaudal direction. All CT examinations were performed without administration of a contrast agent and automatic attenuation-based tube current modulation (CARE dose 4D; Siemens Healthineers) was used.

### CT image reconstruction

Three image sets were acquired in each CT examination: 90 kVp, Sn150 kVp, and weighted average (ratio, 0.5:0.5) to resemble contrast properties of single-energy 120-kVp images. For phantomless volumetric BMD assessment, image series (axial, coronal, and sagittal: section thickness 1 mm, increment 0.75 mm) were reconstructed with a dedicated dual-energy bone kernel (Br69f). The image series were automatically transferred to the picture archiving and communication system (PACS; General Electric Company).

### BMD assessment from lumbar vertebrae

**Fig. 2:**
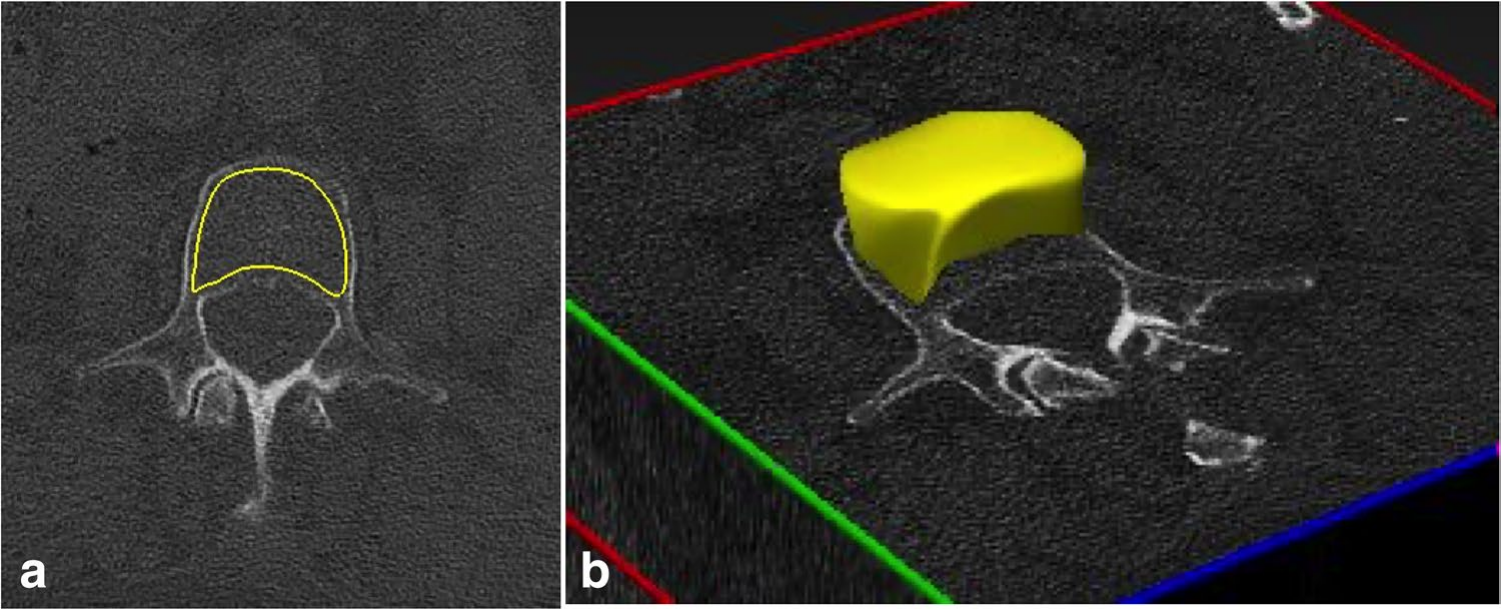
Manual definition of the trabecular volume of interest (VOI) by one reader (C. Booz, radiology resident with 4 years of experience in musculoskeletal imaging) using specific DECT postprocessing software (LiverLab; Fraunhofer IGD) (**a**). This was repeated throughout the entire stack of 2D slices for each vertebra. The resulting three-dimensional VOI (**b**) served as input for volumetric DECT BMD assessment in combination with the two DECT series (90 and Sn150 kVp). VOI, volume of interest; DECT, dual-energy CT; BMD, bone mineral density

Delineation of the trabecular volume of interest (VOI) was required before phantomless volumetric BMD assessment of L1. This was manually performed by one reader (C. Booz, radiology resident with 4 years of experience in MSK imaging) using specific software (LiverLab; Fraunhofer Institute for Computer Graphics Research) (Fig. 2). To define the VOI, one DECT series was uploaded in the software. The reader was then able to define the VOI in 3D, which, in this study, consisted of trabecular bone but not cortical bone of the whole vertebral body. The VOI and the two DECT series (90 and Sn150 kVp) were used as input for volumetric BMD assessment, which was performed using a second software tool (BMD Analysis; Fraunhofer Institute for Computer Graphics Research). The software uses dedicated material decomposition to differentiate collagen matrix, calcium hydroxyapatite, water, fat marrow, and adipose tissue for each voxel, which has been proposed by Nickoloff et al. [8]. In this study, we used an algorithm based on a biophysical model accounting for the five major substances of the trabecular bone:

1 $${{\rm X}^{90}}_{HU} = \left({\mu }^{90}-{\gamma }^{90} g\right) \cdot {V}_{TB} + ({\beta }^{90}t-{\gamma }^{90}g)\cdot {V}_{F}+{\gamma }^{90}g+\delta ,$$

2 $${{\rm X}^{15}}_{0HU} = ({\mu }^{150} - {\gamma }^{150} g) \cdot {V}_{TB} + ({\beta }^{150}t-{\gamma }^{150}g)\cdot {V}_{F}+{\gamma }^{150}g+\delta$$

The HU intensities of both image data sets obtained at 90 and 150 kV (Χ^90^ and Χ^150^) are linked to the fraction of the volume occupied by the matrix material (bone mineral + collagen) V_TB_ and the volume of adipose tissue V_F_. The values for t and g are 0.92 and 1.02, respectively and the other variables represent energy-related constants. Therefore, by calculation of the mean intensity for a region of the trabecular bone in both image data sets, values for V_TB_ and V_F_can be obtained and the BMD value ρBM given in g/cm^3^ can be derived from V_TB_:

*ρ*BM = $\frac{l \bullet {V}_{TB} }{1 + \lambda }$ with l = 3.06 g/cm^3^ and λ = 2.11 being material constants.

Prior to BMD assessment of our study cohort, we randomly selected a sub-cohort of 10 patients and repeated manual delineation 5 times. Variability between the obtained measurements was less than 5% for all selected patients. Consequently, we forewent repeated delineations for each individual vertebra included in the study.

### Statistical analysis

Statistical analysis was performed with dedicated commercial software (Prism 9 for macOS, version 9.0.0, GraphPad Software LLC; MedCalc for Windows, version 13, MedCalc). The difference in baseline characteristics was assessed by unpaired *t*-tests for continuous variables and Fisher’s exact test for categorical values. Receiver operating characteristic (ROC) curve analysis and calculation of the area under the curve (AUC) with Youden’s *J* statistic were performed to evaluate optimal cut-off values for distinguishing BMD values with increased 2-year fracture rates. Associations of patient age, sex, and BMD with the occurrence of follow-up fractures were assessed by logistic regression analysis and goodness of fit was evaluated using Nagelkerke’s *R*^2^. A *p* value less than 0.05 was regarded as statistically significant.

## Results

A total of 261 consecutive patients who had undergone non-contrast third-generation dual-source DECT of the lumbar spine between January 2016 and December 2018 were considered for inclusion in this study. Seventy-one patients were excluded due to suspected or known malignancy, 26 patients due to spondylitis or spondylodiscitis, 32 patients due to the presence of metallic implants following spinal surgery, and 40 patients due to previous fracture or severe deformity of L1. Thus, a total of 92 patients (46 male and 46 female) were ultimately included in this study (Fig. 1). Of these, 50 examinations were performed to rule out suspected fractures, 36 examinations were performed due to chronic pain and 6 examinations were performed to rule out rheumatic lesions of the spine.

**Fig. 3:**
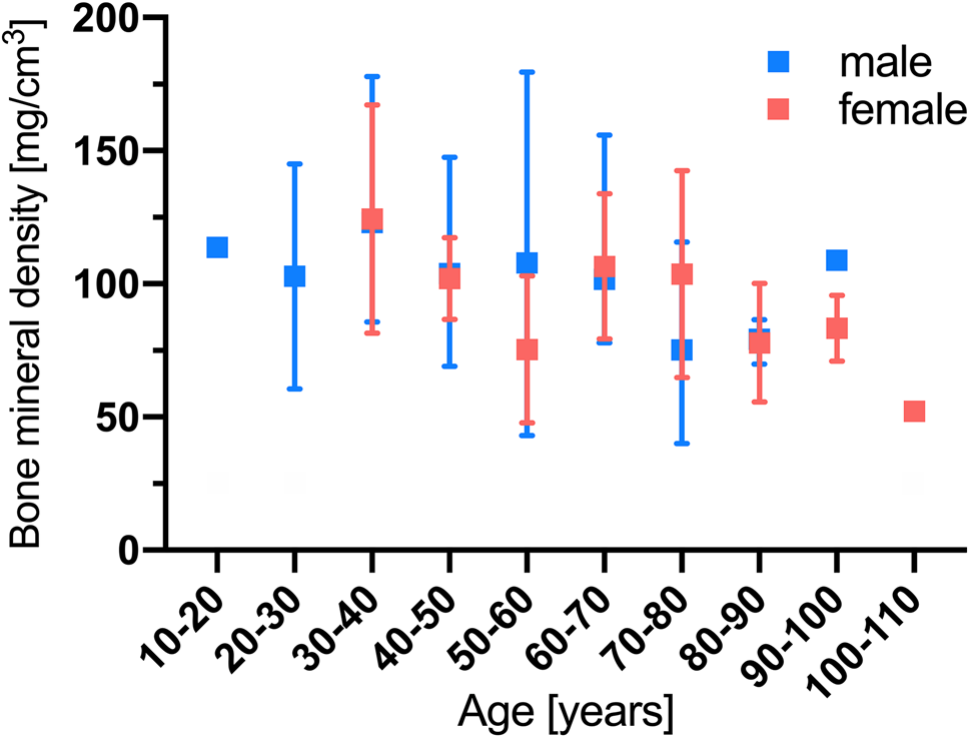
DECT-based BMD values in this study. No significant differences were observed between male and female sex in the same age group (*p* = .208). DECT, dual-energy CT; BMD, bone mineral density

Fifty-five patients (60%) sustained one or more osteoporosis-associated fractures following a non-adequate low-energy trauma within 2 years after DECT and 37 patients (40%) did not sustain an osteoporosis-associated fracture. Fractures affected the vertebral body (*n* = 50, 87.7%), femoral neck (*n* = 4, 7.0%), or humerus (*n* = 3, 5.3%). Patients who sustained one or more fractures were significantly older with a mean age of 69.2 ± 16.7 years compared to patients without fracture (mean age, 52.4 ± 18.3 years). The average time until an osteoporosis-associated fracture was sustained was 72.6 days (± 88.4 days). No significant difference in sex distribution was observed (Fig. 3). Seven patients from the fracture group and 0 patients from the control group had previous DXA-derived BMD measurements with a T-score ≤  − 2.5 indicating osteoporosis. Detailed patient characteristics are shown in Table 1.

**Table 1:** Characterization of the patient population

	Total (*n* = 92)	No fracture (*n* = 37)	Fracture (*n* = 55)	*p* value
Age (years)	63.5 ± 18.8	52.4 ± 18.3	69.2 ± 16.7	< .001
Sex (*n*)				.208
Male	46 (50%)	22 (59,46%)	24 (43,54%)	
Female	46 (50%)	15 (40,54%)	31 (56,36%)	
BMD by DECT [mg/cm^3^]	95.4 ± 33.0 [range, 40.1–179.6]	123.9 ± 28.8 [range, 85.5–179.6]	76.3 ± 18.7 [range, 40.1–108.9]	< .001
Underlying medical conditions or drug use				
Osteoporosis with T-score ≤ *− *2.5	7	0	7	
Osteopenia with T-score ≤ *− *1.0	0	0	0	
Previous fracture of the spine or the hip	24	3	21	
Renal failure	4	2	2	
Hypothyroidism	16	4	12	
Alcohol dependency	5	1	4	
Smoking	2	2	0	
Long-term administration of PPIs	18	7	11	
Long-term administration of glucocorticoids	10	5	5	
Antihormonal therapy	1	0	1	

Detailed patient characteristics. Mean DECT-derived BMD was significantly higher in the control group (*n* = 37) with a value of 123.9 ± 28.8 mg/cm^3^ (range, 85.5–179.6) compared to patients in the fracture group (*n* = 55) with an average value of 76.3 ± 18.7 mg/cm^3^ (*p* < .001)

*BMD*, bone mineral density; *DECT*, dual-energy CT

### Phantomless BMD assessment

**Fig. 4:**
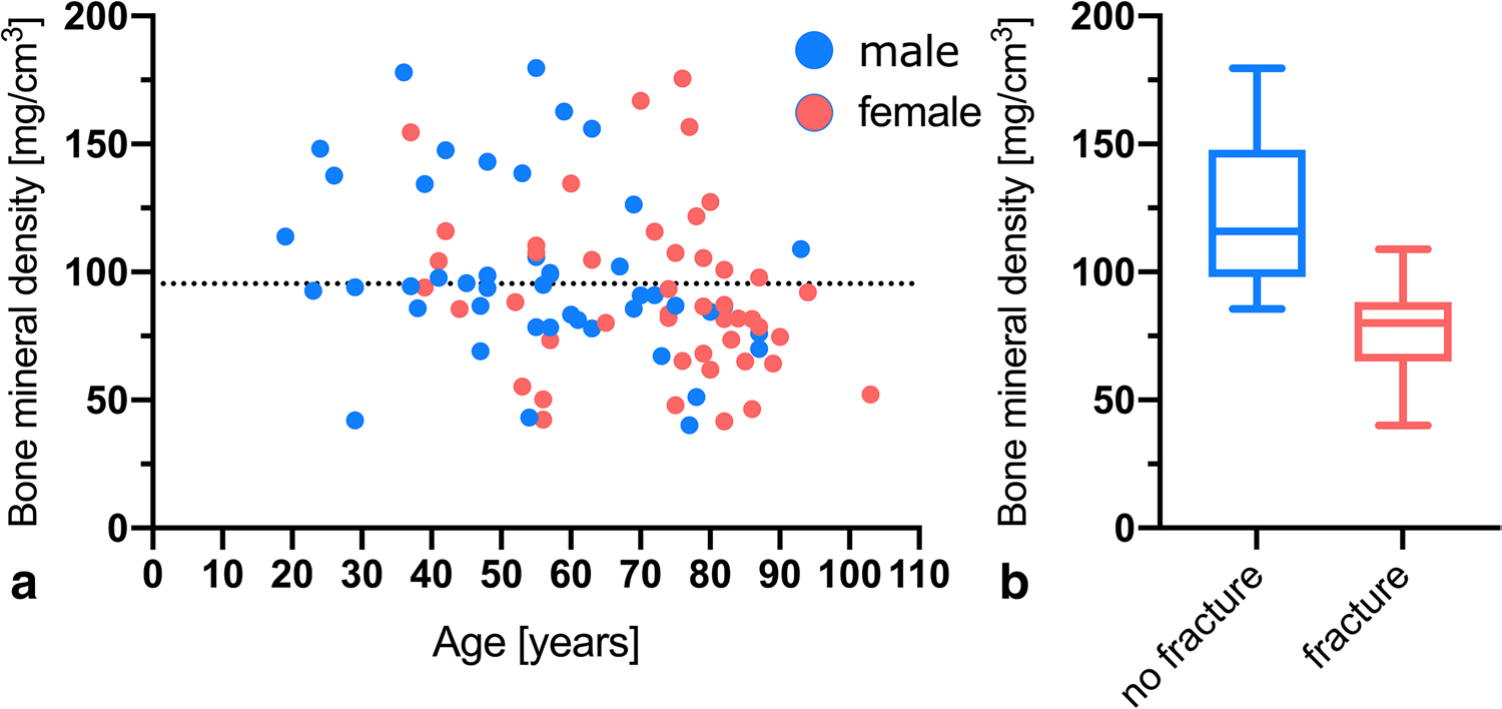
Mean BMD of the study population (**a**) as well as mean BMD of patients with and without incident fractures (**b**). Mean BMD was significantly (*p* < .0001) higher in the control group (123.9 ± 28.8 mg/cm^3^) compared to the fracture group (76.3 ± 18.7 mg/cm^3^). BMD, bone mineral density

Overall mean lumbar DECT-derived BMD value of L1 was 95.4 ± 33.0 mg/cm^3^ (range, 40.1–179.6) (Fig. 4A). Mean DECT-derived BMD was significantly higher in the control group (*n* = 37) with a value of 123.9 ± 28.8 mg/cm^3^ (range, 85.5–179.6) compared to patients in the fracture group (*n* = 55) with an average value of 76.3 ± 18.7 mg/cm^3^ (range, 40.1–108.9) (Fig. 4B).

In line with ACR QCT guidelines, no patient with a fracture during the 2-year follow-up period had a DECT BMD value above 120 mg/cm^3^ and no patient in the control group had a DECT-derived BMD value below 80 mg/cm^3^ [21]. A total of 47 patients had osteopenic DECT-derived BMD values, i.e., values between 80 and 120 mg/cm^3^, 27 (49.1%) patients from the fracture group and 19 (51.4%) patients from the control group.

### Fracture risk prediction

**Fig. 5:**
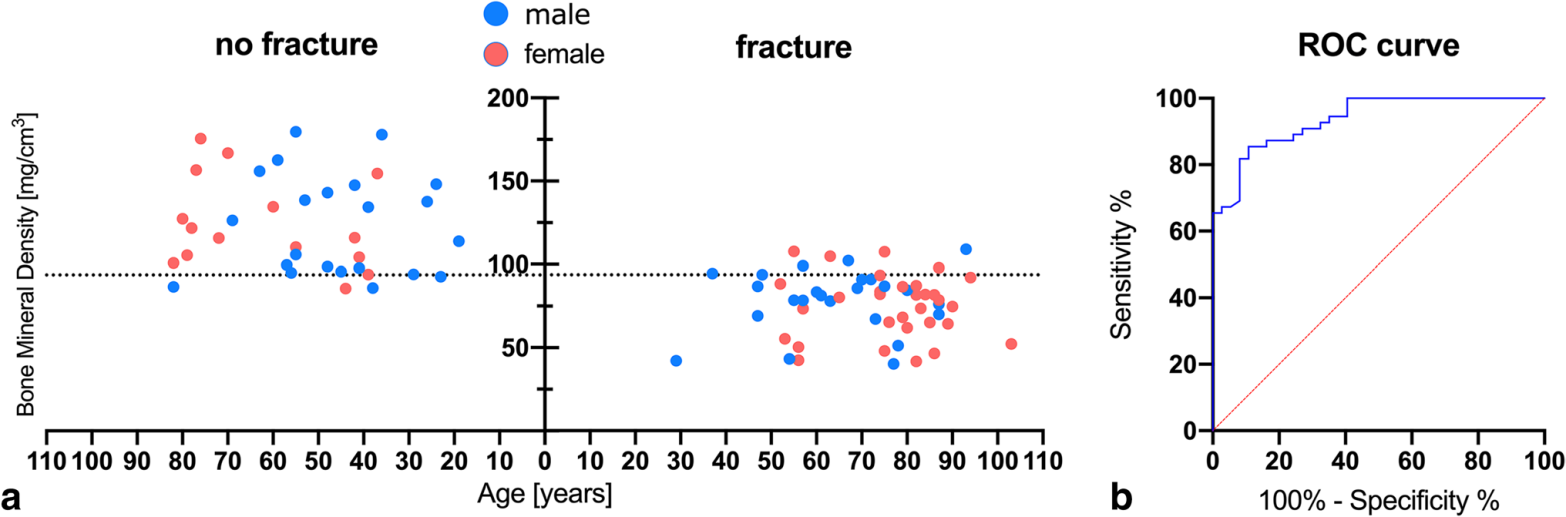
Representative ROC curve analysis of DECT-derived BMD values yields an optimal patient-level cut-off value of 93.7 mg/cm^3^ (**a**) to identify patients at risk of sustaining an osteoporosis-associated fracture within 2 years providing a sensitivity of 85.45%, a specificity of 89.19%, a PPV of 92.2%, and a NPV of 80.5%. AUC was 0.937 (*p* < .0001) (**b**). ROC, receiver operating characteristic; AUC, area under the curve; DECT, dual-energy CT; BMD, bone mineral density; PPV, positive predictive value; NPV, negative predictive value

ROC curve analysis of DECT-derived BMD values yielded an optimal patient-level cut-off value of 93.7 mg/cm^3^ to identify patients at risk of developing a low-energy fracture within 2 years following DECT-based BMD assessment with high sensitivity (85.45% [47/55]), specificity (89.19% [33/37]), PPV (92.2% [45/49]), and NPV (80.5% [33/41]) (Fig. 5A). AUC was excellent with a value of 0.937 (Table 2, Fig. 5B).

**Table 2:** ROC analysis results

Indicator	Value
AUC	0.937 (0.867–0.977)
Optimal threshold	93.7 mg/cm^3^
Sensitivity	85.45% (47/55)
	[73.3–93.5%]
Specificity	89.19% (33/37)
	[74.6–97%]
PPV	92.2% (45/49)
	[82.2–96.8%]
NPV	80.5% (33/41)
	[63.3–88.8%]

ROC curve analysis yields an optimal patient-level cut-off value of 93.7 mg/cm^3^ which shows high diagnostic accuracy for the detection of follow-up fractures in a 2-year interval (*p* < .001). Numbers in square brackets are confidence intervals. *ROC*, receiver operating characteristic; *AUC*, area under the curve; *PPV*, positive predictive value; *NPV*, negative predictive value

### Logistic regression analysis

We performed logistic regression analysis to investigate the relationship of DECT-derived BMD, sex, and age with the occurrence of incidence fractures (Table 3). Increased DECT-derived BMD showed a significant protective effect against the occurrence of new fractures with an odds ratio of 0.8710 (95% CI, 0.091 0.9375, *p* < 0.001) and a significant association of increased age with the incidence of osteoporosis-associated fractures with an odds ratio of 1.0784 (95% CI, 1.0246–1.1350, *p* = 0.004). No significant association between sex and fracture occurrence was observed in our study population. The model fit of the regression model was significant (*p* < 0.001) and showed high goodness of fit with a Nagelkerke *R*^2^ of 0.7899 and ROC-derived AUC of 0.962 (*p* = 0.02).

**Table 3:** Logistic regression analysis

	*β*	*p* value	Odds ratio	95% CI
DECT BMD	− 0.13817	< .001	0.8710	[0.8091;0.9375]
Age	0.075474	< .001	1.0784	[1.0246;1.1350]
Female sex	− 0.039467	0.9627	0.9613	[0.1837;5.0299]
Constant	9.10164	0.0094		

Logistic regression shows a strong protective effect of increased BMD values against the occurrence fractures during a 2-year follow-up interval. Numbers in square brackets are confidence intervals. The regression model is statistically significant with high goodness of fit [χ^2^ = 80.832, *p* < .001], Nagelkerke *R*^2^ = 0.7899, AUC = 0.962. *AUC*, area under the curve; *DECT*, dual-energy CT; *BMD*, bone mineral density

## Discussion

In our study, we showed that BMD measurement derived from phantomless DECT-based volumetric material decomposition of the lumbar spine can accurately predict the occurrence of osteoporosis-associated fractures during a 2-year follow-up period in at-risk patients. A patient-derived optimal BMD cut-off value of 93.7 mg/cm^3^ yielded a sensitivity of 85.45% (CI, 73.3–93.5%), a specificity of 89.19% (CI, 74.6–97.0%), a PPV of 92.2% (CI, 82.2–96.8%), and a NPV of 80.5% (CI, 63.3–88.8%). DECT-derived BMD showed strong associations with the occurrence of fractures during a follow-up of 2 years (OR, 0.871; CI, 0.8091–0.9375).

Osteoporosis and osteoporosis-associated fractures continue to gain more significance as major causes of immobility and frailty in the aging population. Nonetheless, a large gap between expenditure going towards the management and treatment of fractures compared to diagnosis and pharmacological prevention remains. In this context, only 11 patients underwent DXA prior to their trauma which confirmed osteoporosis in 7 cases in our study [1, 2].

With the number of performed CT examinations steadily rising, the opportunistic BMD assessment derived from available imaging data from prior routine examinations may close this diagnostic gap through identification and protection of patients at risk and, simultaneously, reduce radiation exposure and expenditures from frailty fractures. To this end, different methods such as simple HU measurements, phantom-based QCT, CT-based finite element analysis, and dual-layer spectral CT have been proposed for CT-based BMD assessment [22–25]. More recently, other studies showed that HU measurements and asynchronous QCT-derived BMD assessment have the potential to predict osteoporosis-associated fractures, with superior accuracy of QCT-derived BMD measurements [11, 22, 26, 27]. HU measurements and QCT-based BMD assessment, however, are subject to inaccuracies resulting from photoelectric and Compton interactions, tube attenuation voltage levels, scanner settings, and protocols as well as the location of the bones used for assessment [15, 28, 29]. To eliminate many of the previously mentioned distortions, Giambini et al. suggested the use of DECT-based material decomposition to isolate surrounding tissue from bone, thereby allowing more accurate tissue analysis [28].

In our study, we used a dedicated dual-source DECT postprocessing algorithm for phantomless volumetric BMD assessment of the lumbar spine developed by Wesarg et al., which is based on a material decomposition model first introduced by Nickoloff et al. [8, 17]. All imaging data was acquired by the same scanner, using constant settings, protocol, and tube attenuation voltage levels. Therefore, we were able to eliminate almost all previously mentioned confounding factors to achieve comparable and reproducible results. We restricted our analysis to the L1 vertebrae, which has previously been suggested as the optimal target for opportunistic osteoporosis screening from routine CT examinations, since it is included on all abdominal and chest CT examinations, easily identifiable and less prone to degenerative change than other vertebrae. Furthermore, a close correlation has been shown between BMD values obtained from L1 compared to all other lumbar vertebrae, making L1 an ideal surrogate vertebra [25, 30].

In contrast to the established QCT value of 80 mg/cm^3^ according to the ACR, our analysis yielded an optimal DECT-based BMD threshold of 93.7 mg/cm^3^ to distinguish patients who sustained a fracture during a 2-year follow-up period from patients who did not [21, 22]. This finding closely matches previous findings of our group that, contrary to ACR QCT guidelines, identified a DECT-based BMD value of 92 mg/cm^3^ as an optimal cut-off to differentiate osteopenia from normal BMD [18]. This higher threshold can in part be attributed to the removal of aforementioned confounding variables as well as the elimination of technical shortcomings that underestimate conventional QCT-based BMD measurements, such as the fat error [6, 24, 31]. The operating principle of the single-energy technique used in conventional QCT does not allow for material differentiation of bone mass and fatty bone marrow, resulting in underestimation of the bone mass between 7.2 and 25.3% [28, 32]. The algorithm we used identifies the volume of mineralized trabecular bone and directly obtains areal bone mineral density from this volume without the requirement for further corrections by using DECT-based material decomposition [17]. As a result, we were able to minimize accuracy-errors for more accurate assessment of true trabecular BMD, ultimately providing higher sensitivity and specificity for the prediction of fractures than previously reported. In this context, Loeffler et al. showed a sensitivity of 59% and a specificity of 81% for the prediction of vertebral fractures in patients with QCT-derived BMD values below 79.6 mg/cm^3^ [11]. The strong association between DECT-derived BMD and 2-year incidence of frailty fractures was confirmed by logistic regression.

Since routine CT scans are increasingly performed in DECT mode due to several advantages compared to conventional CT, this technique may offer more flexibility in general clinical routine and can help to reduce radiation exposure by avoiding redundant examinations. Furthermore, this technology allows for computation and color-coded display of focal BMD distribution from routine preoperative CT imaging in order to aid in preoperative planning, such as placement of pedicle screws in areas with high stability or to assess the necessity for bone substitute materials during surgery.

This retrospective study has certain limitations that need to be addressed. First, most patients of our study cohort who underwent CT of the spine did so following previous trauma or because of chronic pain. Therefore, due to hospital policy, many of these patients received prior x-ray imaging that could not exclude an acute vertebral fracture and caused a preselection bias towards patients who are at risk to sustain vertebral fractures. Though we only included patients whose imaging data and electronic patient records were available for 2 years or more following DECT of the spine, we cannot exclude the possibility that some vertebral fractures were missed as a result of mild symptoms or due to failed reporting of treatment in other hospitals. Additionally, we sought to eliminate interferences by using only one CT scanner type with a dedicated protocol. Therefore, the applicability of our method with other devices and in the setting of technical heterogeneity has yet to be validated and cut-off values require adjustment in the future.

In conclusion, our study demonstrated that DECT-based BMD assessment can accurately and retrospectively predict the 2-year risk to sustain an osteoporosis-associated fracture in patients at risk without the requirement for a calibration phantom. CT imaging data of the lumbar spine is readily available, especially in elderly patients and patients suffering from osteoporosis. Utilization of this imaging data allows for the early identification of patients at risk to sustain an osteoporosis-associated fracture in the future and, if required, begin treatment without the requirement for redundant examinations. Therefore, DECT-based BMD assessment could help to prevent osteoporosis-associated fractures and ultimately reduce the social and economic burden associated with osteoporosis.

## Abbreviations

ACR: American College of Radiology
AUC: Area under the curve
BMD: Bone mineral density
DECT: Dual-energy computed tomography
DXA: Dual x-ray absorptiometry
HU: Hounsfield unit
QCT: Quantitative computed tomography
ROC: Receiver operating characteristic
ROI: Region of interest
VOI: Volume of interest

## Declarations

### Guarantor

The scientific guarantor of this publication is Prof. Dr. Thomas J. Vogl.

### Statistics and biometry

Prof. Dr. Eva Herrmann kindly provided statistical advice for this manuscript.

### Methodology

•retrospective

## References

[1] Hernlund E, Svedbom A, Ivergård M, et al (2013) Osteoporosis in the European Union: medical management, epidemiology and economic burden: a report prepared in collaboration with the International Osteoporosis Foundation (IOF) and the European Federation of Pharmaceutical Industry Associations (EFPIA). Arch Osteoporos 8: 10.1007/s11657-013-0136-1 10.1007/s11657-013-0136-1 PMC3880487 24113837

[2] Kanis JA, Svedbom A, Harvey N (2014). The osteoporosis treatment gap. J Bone Miner Res.

[3] Jain RK, Vokes T (2017). Dual-energy X-ray absorptiometry. J Clin Densitom.

[4] Schuit SC, van der Klift M, Weel AEA (2004). Fracture incidence and association with bone mineral density in elderly men and women: the Rotterdam Study. Bone.

[5] Bolotin HH (2007). DXA in vivo BMD methodology: an erroneous and misleading research and clinical gauge of bone mineral status, bone fragility, and bone remodelling. Bone.

[6] Yu EW, Thomas BJ, Brown JK (2012). Simulated increases in body fat and errors in bone mineral density measurements by DXA and QCT. J Bone Miner Res.

[7] Fuggle NR, Curtis EM, Ward KA (2019). Fracture prediction, imaging and screening in osteoporosis. Nat Rev Endocrinol.

[8] Nickoloff EL, Feldman F, Atherton JV (1988). Bone mineral assessment: new dual-energy CT approach. Radiology.

[9] Engelke K, Adams JE, Armbrecht G (2008). Clinical use of quantitative computed tomography and peripheral quantitative computed tomography in the management of osteoporosis in adults: the 2007 ISCD official positions. J Clin Densitom.

[10] Li N, Li X, Xu L (2013). Comparison of QCT and DXA: osteoporosis detection rates in postmenopausal women. Int J Endocrinol.

[11] Löffler MT, Jacob A, Valentinitsch A (2019). Improved prediction of incident vertebral fractures using opportunistic QCT compared to DXA. Eur Radiol.

[12] Pickhardt PJ, Pooler BD, Lauder T (2013). Opportunistic screening for osteoporosis using abdominal computed tomography scans obtained for other indications. Ann Intern Med.

[13] Lee HL, Jang JW, Lee SW et al (2019) Inflammatory cytokines and change of Th1/Th2 balance as prognostic indicators for hepatocellular carcinoma in patients treated with transarterial chemoembolization. Sci Rep 9: 10.1038/s41598-019-40078-8 10.1038/s41598-019-40078-8 PMC6397294 30824840

[14] Kim YW, Kim JH, Yoon SH (2017). Vertebral bone attenuation on low-dose chest CT: quantitative volumetric analysis for bone fragility assessment. Osteoporos Int.

[15] Mazess RB (1983). Errors in measuring trabecular bone by computed tomography due to marrow and bone composition. Calcif Tissue Int.

[16] Vetter JR, Perman WH, Kalender WA (1986). Evaluation of a prototype dual-energy computed tomographic apparatus. II. Determination of vertebral bone mineral content. Med Phys.

[17] Wesarg S, Kirschner M, Becker M (2012). Dual-energy CT-based assessment of the trabecular bone in vertebrae. Methods Inf Med.

[18] Booz C, Noeske J, Albrecht MH (2020). Diagnostic accuracy of quantitative dual-energy CT-based bone mineral density assessment in comparison to Hounsfield unit measurements using dual x-ray absorptiometry as standard of reference. Eur J Radiol.

[19] Wichmann JL, Booz C, Wesarg S (2014). Dual-energy CT–based phantomless in vivo three-dimensional bone mineral density assessment of the lumbar spine. Radiology.

[20] Koch V, Müller FC, Gosvig K (2021). Incremental diagnostic value of color-coded virtual non-calcium dual-energy CT for the assessment of traumatic bone marrow edema of the scaphoid. Eur Radiol.

[21] American College of Radiology (2021) ACR–SPR–SSR practice parameter for the performance of musculoskeletal quantitative computed tomography (QCT). Available at: https://www.acr.org/-/media/ACR/Files/Practice-Parameters/qct.pdf?la=en. Accessed 23 Feb 2021

[22] Leonhardt Y, May P, Gordijenko O (2020). Opportunistic QCT bone mineral density measurements predicting osteoporotic fractures: a use case in a prospective clinical cohort. Front Endocrinol (Lausanne).

[23] Allaire BT, Lu D, Johannesdottir F (2019). Prediction of incident vertebral fracture using CT-based finite element analysis. Osteoporos Int.

[24] Roski F, Hammel J, Mei K (2019). Bone mineral density measurements derived from dual-layer spectral CT enable opportunistic screening for osteoporosis. Eur Radiol.

[25] Jang S, Graffy PM, Ziemlewicz TJ (2019). Opportunistic osteoporosis screening at routine abdominal and thoracic CT: normative L1 trabecular attenuation values in more than 20 000 adults. Radiology.

[26] Lee SJ, Graffy PM, Zea RD (2018). Future osteoporotic fracture risk related to lumbar vertebral trabecular attenuation measured at routine body CT. J Bone Miner Res.

[27] Pickhardt PJ, Graffy PM, Zea R (2020). Automated abdominal CT imaging biomarkers for opportunistic prediction of future major osteoporotic fractures in asymptomatic adults. Radiology.

[28] Giambini H, Dragomir-Daescu D, Huddleston PM (2015). The effect of quantitative computed tomography acquisition protocols on bone mineral density estimation. J Biomech Eng.

[29] Knowles NK, Reeves JM, Ferreira LM (2016). Quantitative computed tomography (QCT) derived bone mineral density (BMD) in finite element studies: a review of the literature. J Exp Orthop.

[30] Gerety E-L, Hopper MA, Bearcroft PWP (2017). The reliability of measuring the density of the L1 vertebral body on CT imaging as a predictor of bone mineral density. Clin Radiol.

[31] Bredella MA, Daley SM, Kalra MK (2015). Marrow adipose tissue quantification of the lumbar spine by using dual-energy CT and single-voxel 1 H MR spectroscopy: a feasibility study. Radiology.

[32] Qin L, Huang J, Yu P (2021). Accuracy, agreement, and reliability of DECT-derived vBMD measurements: an initial ex vivo study. Eur Radiol.

